# Effect of bed clay on surface water-wave reconstruction from ripples

**DOI:** 10.1038/s41598-024-78821-5

**Published:** 2024-12-28

**Authors:** Jonathan Malarkey, Ellen M. Pollard, Roberto Fernández, Xuxu Wu, Jaco H. Baas, Daniel R. Parsons

**Affiliations:** 1 School of Ocean Sciences, Bangor University, Menai Bridge, Anglesey, LL59 5AB UK; 2 Energy and Environment Institute, University of Hull, Hull, HU6 7RX UK; 3 Department of Civil and Environmental Engineering, The Pennsylvania State University, University Park, PA 16802 USA; 4 School of Ocean and Earth Science, University of Southampton, Southampton, SO14 3ZH UK; 5 Department of Geography and Environment, Loughborough University, Loughborough, LE11 3TU UK

**Keywords:** Geology, Oceanography, Sediment transport, Sand-clay mixtures, Wave reconstruction, Wave ripples, Ocean sciences, Geomorphology

## Abstract

Wave ripples can provide valuable information on their formative hydrodynamic conditions in past subaqueous environments by inverting dimension predictors. However, these inversions do not usually take the mixed non-cohesive/cohesive nature of sediment beds into account. Recent experiments involving sand–kaolinite mixtures have demonstrated that wave-ripple dimensions and the threshold of motion are affected by bed clay content. Here, a clean-sand method to determine wave climate from orbital ripple wavelength has been adapted to include the effect of clay and a consistent shear-stress threshold parameterisation. From present-day examples with known wave conditions, the results show that the largest clay effect occurs for coarse sand with median grain diameters over 0.45 mm. For a 7.4% volumetric clay concentration, the range of possible water-surface wavelengths and water depths can be reduced significantly, by factors of three and four compared to clean sand, indicating that neglecting clay when present will underestimate the wave climate.

## Introduction

Bed-surface structures in sediments and sedimentary rocks of past subaqueous environments provide important information on flow hydraulics^1^. These structures tend to be classified on the basis of the presence or absence of cohesion in equivalent modern environments: (a) cohesive structures, associated with physical cohesion by clay particles and biological cohesion by extracellular polymeric substances (EPS), e.g. biofilms^2^, where the bed is stabilised by cohesion between grains and sudden catastrophic failure may occur under high bed shear stress, e.g. during storms; and (b) non-cohesive bedforms (e.g. wave and current ripples^3,4^), where grains can move individually and tend to respond more rapidly and continuously to changes in flow forcing^5^. Examples of these types of bed-surface structure include roll-ups^6^ and non-cohesive wave ripples^7^. Davies et al.^8^ argued that the distinction between cohesive and non-cohesive sedimentary structures is artificial, as they represent end members of a spectrum, and thus predictions based on one classification may result in misinterpretations. This position is re-enforced by recent experiments that have shown how sandy bedforms can be affected by small amounts of biological and physical cohesion^9–12^. The consequence for wave ripples of physical cohesion associated with kaolin clay in the bed has been detailed by Wu et al.^13^. Building on previous work^7^, Diem^14^ developed a clean-sand analytical method for the prediction of paleowave climate based on the dimensional measurement of wave ripples in the rock record^15–19^. Here, the Diem^14^ approach is adapted for sand–clay mixtures, using the synthesis proposed by Wu et al.^13^.

The Diem^14^ approach starts by determining the wave orbital diameter from the ripple wavelength, without requiring a specific wavelength predictor. Here, the formulation starts as Diem^14^ did, with linear wave theory and additional constraints based on threshold of motion and wave breaking. It will then return to the effect of clay on ripple-wavelength prediction and the threshold of motion. Since wave conditions from the rock record are unknown, the importance of clay content is demonstrated with the use of present-day examples from the laboratory and field, where the wave conditions were known.

## Results

### The Diem^14^ approach

#### Wave conditions

Based on linear wave theory, the Diem^14^ approach uses expressions for the dispersion relation and the wave-velocity amplitude, *U*_0_, together with conditions for the threshold of motion and wave breaking, to give

1a $$x<\tanh kh,$$

1b $$x \geqslant A\cosh kh,$$

(see “Methods”) where *x* = *L*/*L*_t∞_, *L* is the water-surface wavelength, *L*_t∞_ = π*g*(*d*_0_/*U*_t_)^2^/2 is the deep-water surface wavelength corresponding to the threshold of motion, *k* = 2π/*L*, *h* is the water depth, *g* is the acceleration due to gravity (= 9.81 m s^2^), *d*_0_ is the orbital diameter (= *H*/sinh*kh*, *H* is the wave height), *U*_t_ is the critical wave-velocity amplitude associated with the threshold of motion and *A* = *d*_0_/0.142*L*_t∞_. As will be seen later, *U*_t_ is a function of *d*_0_ and *D*_50_, the median grain diameter, and *A* can be defined as (*U*_t_*/U*_m_)^2^/2, where *U*_m_ = (0.0355π*gd*_0_)^1/2^ is the maximum wave-velocity amplitude. Since wave-velocity amplitude and orbital diameter are related by *U*_0_ = π*d*_0_/*T*, where *T* is the wave period, *U*_0_ and *T* are in the ranges *U*_t_ < *U*_0_ ≤ *U*_m_ and π*d*_0_/*U*_m_ ≤ *T* <  π*d*_0_/*U*_t_.

**Fig. 1:**
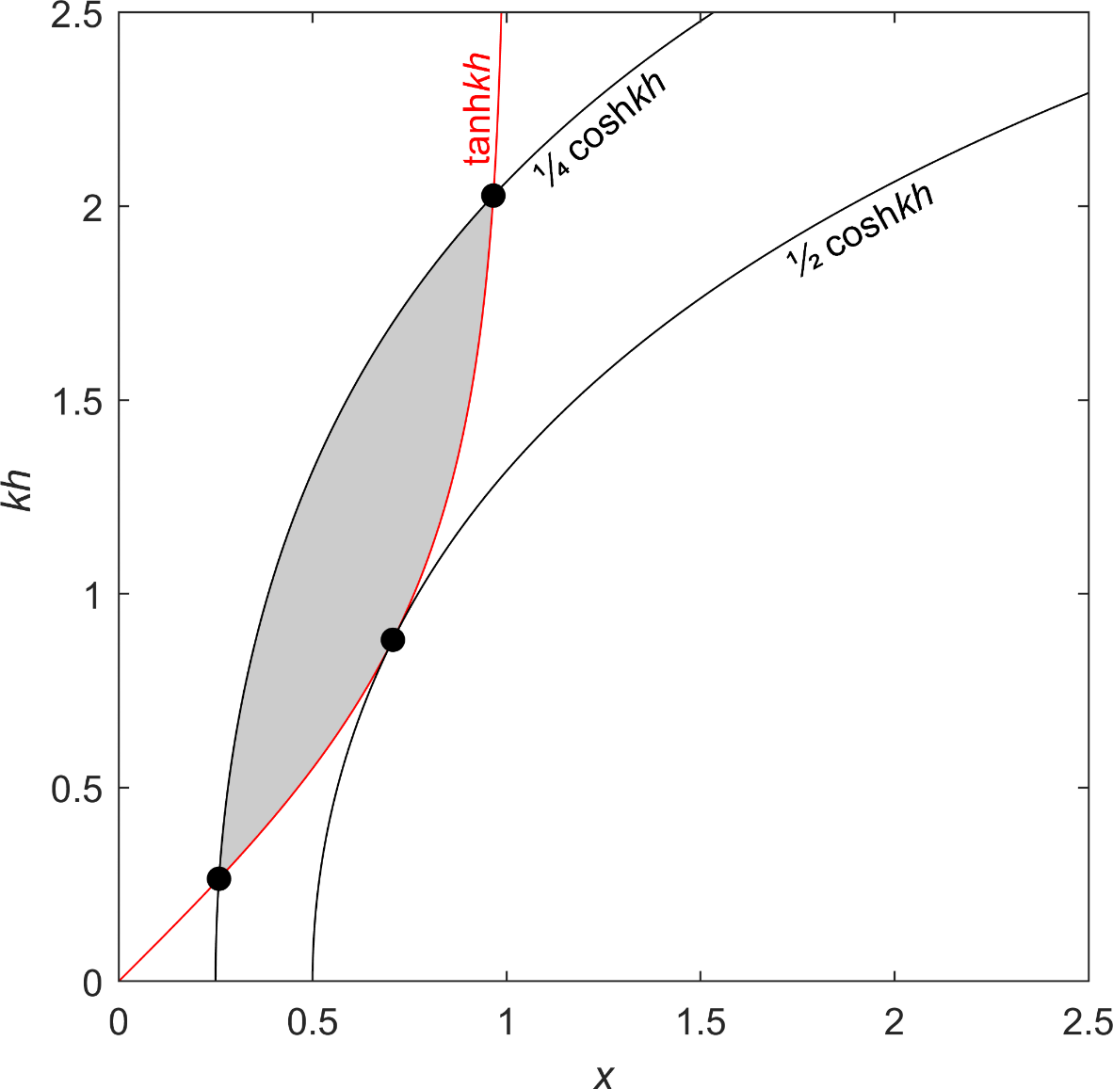
*kh* versus *x* (*L*/*L*_t∞_) for the limiting case of *A* = 1/2 and also *A* = 1/4. The dots correspond to *x* = 2^–1/2^ and *kh* = arctanh2^–1/2^, for *A* = 1/2; *x*_max, min_ = (2 ± 3^1/2^)^1/2^/2 and *kh*_max, min_ = arctanh[*x*_max, min_], for *A* = 1/4 (see “Methods”, Eq. (9)), and shading represents allowable values of *x* and *kh* for *A* = 1/4 (*A*cosh*kh* ≤ *x* < tanh*kh*).

Equation (1a, b) represent the range of possible conditions between threshold and wave breaking for the wave climate, such that *x* is in the range *A*cosh*kh* ≤ *x* < tanh*kh*, and *A* < 1/2 for there to be any allowable conditions (see “Methods”). Figure 1 shows *kh* versus *x* for the limiting single-valued *A* = 1/2 case (*U*_m_^2^ = *U*_t_^2^) from shallow water (*kh* ≪ 1) to deep water (*kh* ≫ 1). Figure 1 also shows the *A* = 1/4 case (*U*_m_^2^ = 2*U*_t_^2^), corresponding to typical above-threshold wave conditions. In the latter case, the shaded region in Fig. 1 shows the allowable values of *x* [the “Methods” section explains how intersection points, (*x*_min_, *kh*_min_) and (*x*_max_, *kh*_max_), are determined]. The breaking-wave curves (*x* = *A*cosh*kh*) are concave downward and the threshold curve (*x* = tanh*kh*) is concave upward. Notice for the breaking-wave curves, *x* → *A* for *kh* ≪ 1 and *A* also controls the slope for larger *kh*. In dimensional terms, *L* is therefore limited by the threshold scale (*L*_t∞_) and breaking-wave scale (*AL*_t∞_) according to *AL*_t∞_ < *L* ≤  *L*_t∞_. Then, from equations (1a, b), the range of *h* can be expressed as a function of *L* as.

2 $$(L/2\pi ){\text{arctanh}}(L/{L_{{\text{t}}\infty }})<h \leqslant (L/2\pi ){\text {arccosh}}(L/A{L_{{\text{t}}\infty }})$$

#### The ripple predictor

Diem’s^14^ central assumption is that the orbital diameter can be expressed in terms of a ripple wavelength, which is assumed to be in equilibrium, λ_e_, as

3 $${\uplambda _{\text{e}}}={\upalpha _0}{{\text{d}}_0},$$

where α_0_ = 0.65, based on the experiments^20^, provided that λ_e_ < 200 mm (*d*_0_ = 308 mm). Above this limit, Diem^14^ used the Sleath^21^ predictor. This arbitrary 200-mm limit represents the lower boundary of the suborbital and anorbital ranges, where the wavelength is dependent on both *d*_0_ and *D*_50_ for suborbital ripples and only dependent on *D*_50_ for anorbital ripples. However, while a value of α_0_ in the range 0.5 ≤ α_0_ ≤ 0.75 in Eq. (3) is widely accepted, there is little agreement in the literature on the precise nature of the orbital, suborbital, and anorbital limits^22^. Wiberg and Harris^3^ defined orbital, suborbital and anorbital ripples by *d*_0_/*D*_50_ ≤ 1754, 1754 < *d*_0_/*D*_50_ ≤ 5587 and *d*_0_/*D*_50_ > 5587, respectively^23^, whereas other researchers have argued the anorbital limit should have wave-period dependence^24,25^. Provided that Eq. (3) *does* hold, which is what will be assumed here, all quantities involving *d*_0_ can now be expressed in terms of λ_e_. Wu et al.^13^, and other researchers previously^26,27^, have demonstrated that *d*_0_ can be modified by the presence of a current. However, as the method relies on there being a one-to-one correspondence between *d*_0_ and λ_e_, this effect cannot be included and so conditions must be restricted to waves in isolation.

### Adaptions to the Diem^14^ approach

#### Threshold of motion parameterisation

Based on the Soulsby^28^ critical threshold of motion for clean sand, the “Methods” section derives an expression for *U*_t_^2^, Eq. (11), such that *U*_t_^2^, *L*_t∞_ and *AL*_t∞_ can be written

4a $$U_{\text{t}}^{{~2}}=~B(gd_{0}^{{~0.52}}D_{{50}}^{{~0.48}}),$$

4b $${L_{\text{t}\infty }}~=\frac{{\uppi {d_0}}}{{2B}}{\left( {\frac{{{d_0}}}{{{D_{50}}}}} \right)^{0.48}},$$

4c $$A{L_{\text{t}\infty }}~=\frac{{{d_0}}}{{0.142}},$$

where *B* = 3.653(*s*–1)θ_0_, *s* is the relative density of sediment in water, θ_0_ is the critical skin friction Shields parameter, Eq. (10), and *d*_0_ = λ_e_/α_0_ from Eq. (3). Diem^14^ used the Komar and Miller^29^ mobility threshold prescription. Here, the Soulsby^28^ expression has been used, as it allows *U*_t_^2^ to be directly related to θ_0_ and it avoids the need for two different functional forms, for *D*_50_ < 0.5 mm and *D*_50_ ≥ 0.5 mm (Eqs. (12) and (13)).

#### Inclusion of the effect of clay

Wu et al.^13^ showed that the ratio of wavelength to orbital diameter, α, which replaces α_0_ in Eq. (3), can be expressed as

5 $${\upalpha}\,=\,{\upalpha_0}\left\{ {\begin{array}{*{20}{l}} {1,}\quad\quad\quad\quad\quad\quad\quad\quad\quad{{C_0}}\leqslant{C_{0{\text{m}}},} \\ {1-5.5}({C_0}-{C_{0{\text{m}}}),}\quad{C_0} >{C_{0{\text{m}}},} \end{array}} \right.$$

where α_0_ is the clean-sand constant of proportionality (= 0.61), *C*_0_ is the clay content in the bed, *C*_0m_ = 7.4% is the minimum value of *C*_0_ where α can change from α_0_ and α = α_0_/2 for *C*_0_ = 16.3%. Whitehouse et al.^30^ showed that the threshold of motion is enhanced by the clay content according to

6 $${\uptheta_{0\text{E}}}={\uptheta _0}{B_\theta },$$

where θ_0_ is the clean-sand threshold, *B*_θ_ = 1 + *P*_θ_*C*_0_ and *P*_θ_ is a constant that depends on the sediment properties. Based on their experiments, Wu et al.^13^ determined that *P*_θ_ = 6.3 for *D*_50_ = 0.143 mm and *P*_θ_ = 23 for 0.45 ≤ *D*_50_ ≤ 0.5 mm (between these two ranges, it will be assumed that *P*_θ_ can be linearly interpolated). Notice in Eq. (6) that even small amounts of clay produce an enhancement which is strongly dependent on grain size.

Thus, the two main effects of including clay are that α is reduced and θ_0*E*_ is increased. Substituting Eqs. (5) and (6), into Eqs. (4a, b, c) gives *U*_t_^2^, *L*_t∞_ and *AL*_t∞_ as

7a $$U_{\text{t}}^{{~2}}=~B\left[ {\frac{{{B_\theta }}}{{{\upalpha ^{0.52}}}}} \right](g\uplambda _{\text{e}}^{{0.52}}D_{{50}}^{{~0.48}}),$$

7b $${L_{\text{t}\infty }}~=\frac{{\pi {\uplambda _{\text{e}}}}}{{2B[{B_\theta }{\upalpha ^{1.48}}]}}{\left( {\frac{{{\uplambda _{\text{e}}}}}{{{D_{50}}}}} \right)^{0.48}},$$

7c $$A{L_{\text{t}\infty }}~=\frac{{{\uplambda _{\text{e}}}}}{{0.142[\upalpha ]}},$$

where λ_e_ is the mixed clay–sand ripple wavelength and only the square-bracketed quantity in each expression depends on *C*_0_.

#### The adapted procedure

The procedure begins with the determination of the ripple wavelength, λ_e_, and bed-clay content, *C*_0_. Once these have been determined, the following calculations are undertaken:

(i) Use λ_e_ and *C*_0_ in equations (7a, b,c), with α and *B*_θ_ given by Eqs. (5) and (6), to determine *U*_t_, *L*_t∞_ and *AL*_t∞_
(ii) Use *A* in Eq. (9) to determine *x*_max, min_, so that *x*_min_ ≤ *x* ≤ *x*_max_
(iii) Use *L* = *L*_t∞_*x* to determine the range of *h* based on Eq. (2) and *U*_t_ and *U*_m_ = (0.0355π*g*λ_e_/α)^1/2^ to determine the ranges of *U*_0_ and *T*: *U*_t_ < *U*_0_ < *U*_m_ and πλ_e_/α*U*_m_ < *T * <  πλ_e_/α*U*_t_

#### Example cases

With specific examples from the rock record, Diem^14^ was able to show how local considerations and context could be used to further limit the theoretical ranges described in the previous section. Here, modern-day examples, where the wave properties are known, are used, so that attention can be focussed on the effect of clay on the theoretical ranges alone. The example cases correspond to clean, coarse-, medium- and fine-grained sand from the laboratory and field, and involve determining how wave conditions based on the measured ripples change if the clay content is varied in the range 0 ≤ *C*_0_ ≤ 16.3%.

#### Wu et al.^11^, coarse-sand laboratory data

**Fig. 2:**
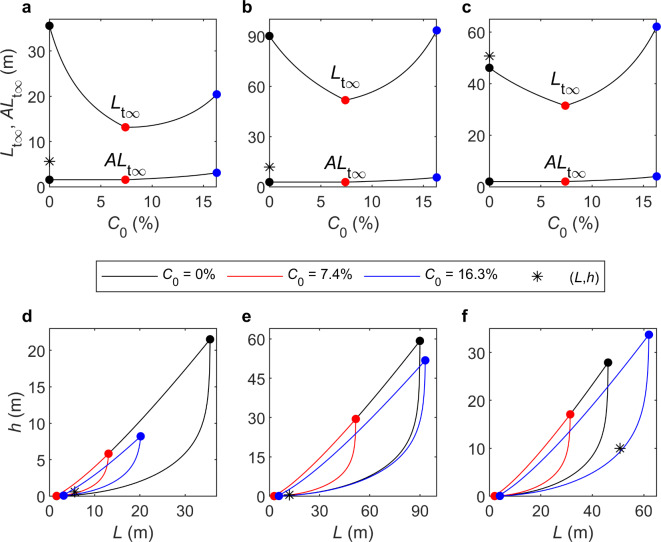
(**a**–**c**) Threshold, *L*_t∞_, and wave-breaking, *AL*_t∞_, scales, equations (7b, c), versus *C*_0_ and (**d**–**f**) *L*–*h* phase space from Eq. (2), showing the different ranges for *C*_0_ = 0, 7.4 and 16.3% and the measured *L* and *h*. For (**a**,**d**) Wu et al.^11^, λ_e_ = 278*D*_50_, *D*_50_ = 0.496 mm, θ_0_ = 0.032, *L* = 5.62 m and *h* = 0.6 m; for (**b**,**e**) Doucette^32^, λ_e_ = 250 mm, *D*_50_ = 0.22 mm, θ_0_ = 0.045, *L* = 11.9 m and *h* = 0.47 m, and for (**c**,**f**) Boyd et al.^33^, λ_e_ = 180 mm, *D*_50_ = 0.11 mm, θ_0_ = 0.076, *L* = 50.7 m and *h* = 10 m. Legend applies to (**d**–**f**); colours in (**a**–**c**) are consistent with the legend.

Wu et al.^11^ conducted a series of experiments involving a single-wave condition over a bed composed of well-sorted coarse sand, *D*_50_ = 0.496 mm (θ_0_ = 0.032), and varying clay content, 0 ≤ *C*_0_ ≤  7.4%. For the clean sand experiment (*C*_0_ = 0%), the wave conditions were given by *h* = 0.6 m, *H* = 0.16 m and *T* = 2.49 s (*L* = 5.62 m), corresponding to *d*_0_ = 0.223 m. Despite the coarse grain size, which allowed clay and sand particles to be distinguished more readily in the experiments, Wu et al.^12^ demonstrated that these hydrodynamic conditions were comparable to an intertidal site in the macrotidal Dee Estuary with a medium grain size^31^. This experiment produced ripples with a wavelength λ_e_ = 278*D*_50_^13^. Figure 2a shows the threshold and wave-breaking scales, *L*_t∞_ and *AL*_t∞_, versus *C*_0_. *L*_t∞_, which is smallest at *C*_0_ = 7.4%, has a much larger range than *AL*_t∞_, which is constant for *C*_0_ ≤  7.4% and then doubles up to *C*_0_ = 16.3%. Figure 2d shows the corresponding *L*-*h* phase space, based on Eq. (2), for the clay contents depicted in Fig. 2a (*C*_0_ = 0, 7.4 and 16.3%). Compared to the dimensionless *x*–*kh* plot (Fig. 1), the threshold curves are still concave downwards, but more exaggerated, and the breaking-wave curves are close to straight lines. The change in ranges is largely due to changes in *L*_t∞_. The reduction in range between the largest and smallest (corresponding to *C*_0_ = 0% and 7.4%) is by a factor of 3 and 4 for the water-surface wavelength and water depth, respectively (Fig. 2d). Notice that the actual surface wavelength and water depth (*L* = 5.62 m, *h* = 0.6 m) are within all three ranges. Figure 2a, d can be compared with Fig. 4a, b to see the effect of using the Komar and Miller^29^ clean-sand mobility description for the threshold. This shows *L*_t∞_ to be about 63% of its value in Fig. 2a, because *B* = 0.21(*s*–1) = 0.34 as opposed to 3.653(*s*–1)θ_0_ = 0.19. Thus, using the Diem^14^ clean-sand mobility description underpredicts the range of water-surface wavelengths and heights in an absolute sense. In a relative sense, the change in the ranges with clay content is similar, because the powers of *d*_0_ and *D*_50_ are similar (Eq. (13), for *D*_50_ < 0.5 mm, and Eq. (7a)), but this will not be the case for *D*_50_ > 0.5 mm. Also, the measured *L* and *h* are not within the *C*_0_ = 7.4% range (Fig. 4b). As *L* and *h* are below the threshold curve, this would imply that ripples of this size are relict for this clay content. This is inconsistent with the experimental results, since Wu et al.^13^ showed no reduction in λ_e_ for *C*_0_ ≤  7.4%.

#### Doucette^32^, medium-sand field data

The Doucette^32^ field measurements were taken on a microtidal beach of Wambro Sound, Western Australia, near Perth (run 1) where *h* = 0.47 m, *H* = 0.2 m and *T* = 5.6 s (*L* = 11.9 m), corresponding to *d*_0_ = 0.79 m. The bed was composed of medium sand, with *D*_50_ = 0.22 mm (θ_0_ = 0.045), and the measured ripples had a wavelength of *λ*_e_ = 250 mm. This example case builds on the Wu et al.^11^ example case by corresponding to both the hydrodynamics and medium grain size of the Dee Estuary UK^31^. Since *d*_0_/*D*_50_ = 3591, the ripples were in the suborbital range, where the wavelength is dependent on both the orbital and grain diameters. Notice this wavelength is above Diem’s^14^ 200-mm limit. The methods section demonstrates that, whilst using the Sleath^21^ predictor for *C*_0_ = 0 produces a difference, it is similar to the other two example cases, which are below the Diem^14^ limit, and so is not considered significant. From interpolation, *P*_θ_ in equation (6) is determined to be 10. Figure 2b shows *L*_t∞_ and *AL*_t∞_ versus *C*_0_ and Fig. 2e shows the *L–h* phase space, for *C*_0_ = 0, 7.4 and 16.3%. These reveal similar behaviour to that of the coarse-grained sand case, but less extreme: in Fig. 2b, *L*_t∞_ is still at its minimum at *C*_0_ = 7.4% and *AL*_t∞_ shows the same enhancement as in Fig. 2a. The reduction in range between the largest and smallest (*C*_0_ = 0% and 7.4%) is by a factor of 2 for both the water-surface wavelength and water depth (Fig. 2e). Again, the actual surface wavelength and water depth (*L* = 11.9 m, *h* = 0.47 m) are within all three ranges.

#### Boyd et al.^33^, fine-sand field data

The Boyd et al.^33^ field measurements were undertaken about 1 km from Martinique Beach on the Atlantic coast of Nova Scotia during a period of relative calm (day 167, hour 9) where *h* = 10 m, *H *= 0.5 m and *T* = 6.2 s (*L* = 50.7 m), corresponding to *d*_0_ = 0.32 m. The bed was composed of well-sorted fine sand, with *D*_50_ = 0.11 mm (*θ*_0_ = 0.076), and the measured ripples had a wavelength of *λ*_e_ = 180 mm. Fine sands are common in many estuaries around the world, for example^34^. *d*_0_/*D*_50_ = 2873 puts the ripples into the suborbital range. Assuming that Pθ in equation (6) is the same as for 0.143 mm (*P*_θ_ = 6.3), Fig. 2c shows *L*_t∞_ and *AL*_t∞_ versus *C*_0_ and Fig. 2f shows the L–h phase space, for *C*_0_ = 0, 7.4 and 16.3%. *L*_t∞_ in Fig. 2c is still at its minimum at *C*_0_ = 7.4%, but, because of far weaker clay enhancement of the threshold for fine sands in equation (6), *L*_t∞_ is largest for *C*_0_ = 16.3%. In Fig. 2f, the measured water-surface wavelength and water depth (*L* = 50.7 m, *h* = 10 m) are below the threshold curve and outside the range for the *C*_0_ = 0 and 7.4% clay contents, and just above the threshold curve and within range for *C*_0_ = 16.3%, because, unlike the previous cases, *C*_0_ = 16.3% produces the largest *L*_t∞_. Since there was little clay at the field site, the wave conditions were probably below threshold, implying that the observed ripples were relict. This is supported by the fact that Boyd et al.’s^33^ previous observation at day 167, hour 3, showed the same wavelength and no ripple migration. The reduction in range between the largest and smallest (*C*_0_ = 16.3% and 7.4%) is again by a factor of 2 for both the water-surface wavelength and water depth (Fig. 2f).

## Discussion

**Fig. 3:**
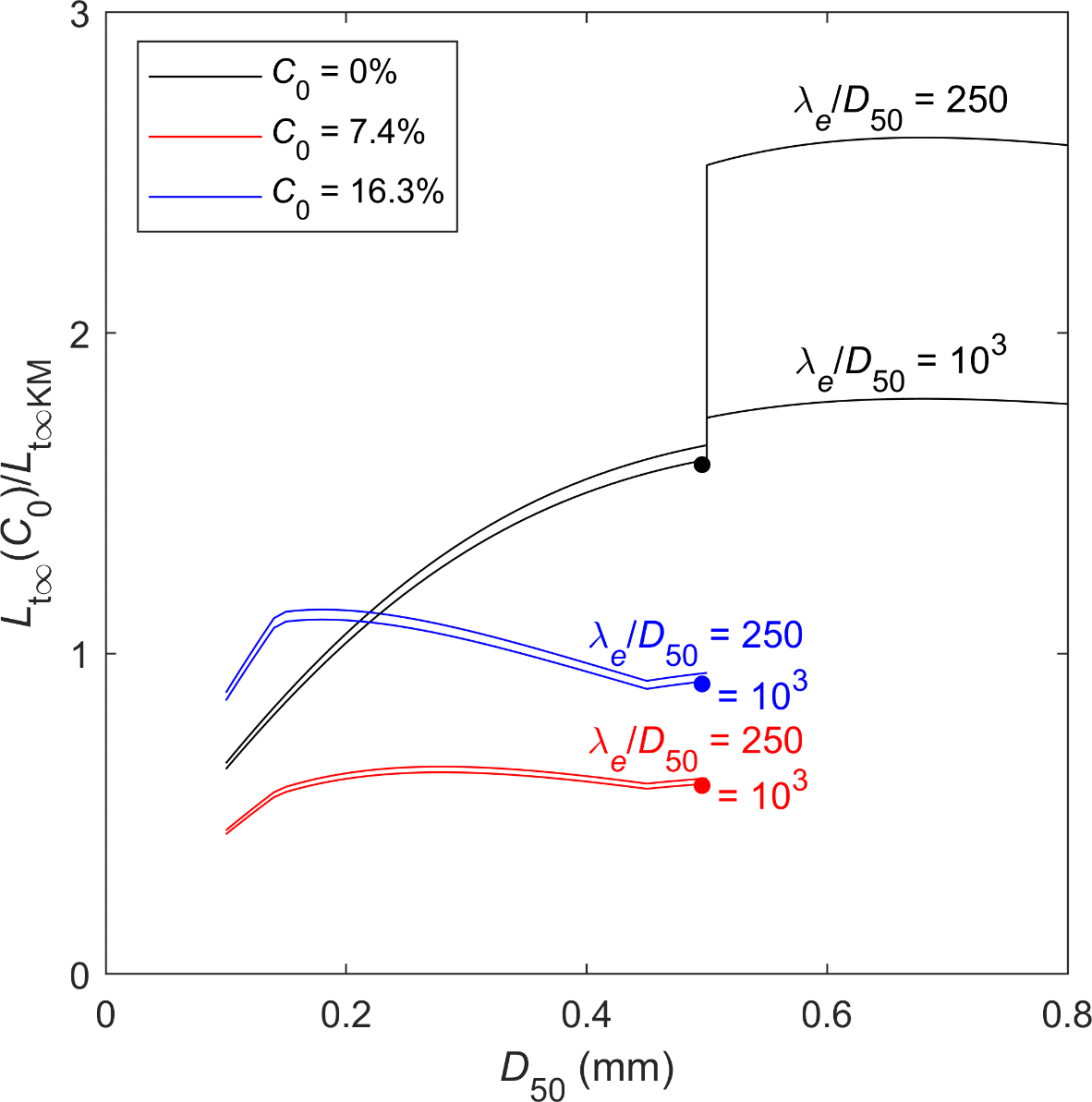
Relative size of *L*_t∞_(*C*_0_) from Eq. (7b) normalised by the clean-sand *L*_t∞_ from Eq. (13), *L*_t ∞ KM_, for *C*_0_ = 0, 7.4 and 16.3%. λ_e_/*D*_50_ = 250 and 1000, and the dots correspond to the Wu et al.^11^ clean-sand experiment in Fig. 2a.

The range of *L* shown in Fig. 2 is largely controlled by *L*_t∞_, so it is of interest to determine how the change in clay content affects *L*_t∞_, Eq. (7b), compared to the original clean-sand Diem^14^ method using Komar and Miller^29^, *L*_t∞KM_, Eq. (13) with *C*_0_ = 0%. The net effect is shown as a ratio in Fig. 3 for *C*_0_ = 0, 7.4 and 16.3% and 0.1 ≤ *D*_50_ ≤ 0.8 mm, for the approximate limits in the range of λ_e_/*D*_50_ of 250 and 1,000. There are two competing effects: the reduction because of clay content (Fig. 2) and the increase because of using the Soulsby^28^ threshold condition rather than the Komar and Miller^29^. Figure 3 shows a discontinuity for clean sand at *D*_50_ = 0.5 mm as a result of Eq. (13), leading to the largest difference (*L*_t∞_ is increased by up to 161% for λ_e_/*D*_50_ = 250), which decreases with increasing λ_e_/*D*_50_ (although the Diem^14^ method has rarely been applied for *D*_50_ > 0.5 mm). Otherwise for *D*_50_ ≤ 0.19 mm, *L*_t∞_ is reduced by up to 36%, and for 0.19 < *D*_50_ ≤ 0.5 mm, *L*_t∞_ is increased by up to 64%. For *C*_0_ = 7.4%, *L*_t∞_ is consistently decreased by between 35 and 56%, and for *C*_0_ = 16.3%, *L*_t∞_ varies only slightly (increased by up to 14%, for 0.12 ≤ *D*_50_ ≤ 0.37 mm, and otherwise reduced by up to 15%). The absence of a discontinuity in the present formulation, compared to Diem’s^14^ original formulation, is clearly preferable. Also, the net effect of the clay on *L*_t∞_ will be stronger for smaller than for larger clay contents. It should be remembered that the present method, like the original Diem^14^ method, is only applicable to waves alone.

It is important to clarify how a representative clay content, *C*_0_, for the ripples should be determined. In the modern environment this usually involves measuring *C*_0_ below the active layer (below trough level), as efficient winnowing often removes clay from the body of the ripples during development^12^. In the geological record, where the method still requires testing, clay content in deposits should be based on primary clay minerals and diagenetic alterations for which it can be established that the original mineral was part of the primary clay fraction. The laboratory experiments used in this study^13^ are based only on the physical cohesion associated with kaolin, and not the biological cohesion associated with EPS that often occur together with clay in the field^35^. Since, the effect of EPS can be quite substantial^2,9^, the present method should be interpreted as a conservative estimate in the field. For instance, Baas et al.^35^ concluded that ripple development in the field compared to the laboratory was significantly delayed due to the additional presence of EPS leading to a greater enhancement in the threshold of motion, see Eq. (6). EPS delaying development may also challenge the equilibrium wavelength assumption, see Eq. (3). Further study of the effects of combined clay and EPS on wave ripples is thus still required.

## Conclusions

Preserved sedimentary bedforms provide important information for reconstructing past hydraulics in subaqueous environments by inverting bedform predictors, but this is usually based exclusively on non-cohesive sand. The present work incorporates the effects of sand-clay mixtures on bedforms, using the experimental results of Wu et al.^13^ in the non-cohesive inversion method of Diem^14^ for waves alone. Based on wave breaking and threshold of motion limitations, the Diem^14^ approach results in ranges for wave conditions. Here we have shown that the inclusion of as little as 7.4% clay in the most extreme case of coarse sand, *D*_50_ ≥ 0.45 mm, reduces the possible ranges of water-surface wavelengths and water depths by factors of 3 and 4, respectively. For fine sand, the ranges are reduced by a factor of two. In short, not accounting for the modifying effect of clay in ripple growth and equilibrium geometries, may lead to underestimating the prevailing wave conditions if clay is present.

## Methods

### Derivation of the Diem^14^ approach

For linear wave theory, the dispersion relation and the wave-velocity amplitude, *U*_0_, are

8a $${\upsigma ^2}=gk\tanh kh,$$

8b $${U_0}=\uppi {d_0}/T,$$

where σ = 2π/*T*, *d*_0_ = *H*/sinh*kh* and *k* = 2π/*L*. The wave properties, characterised by equations (8a, b), are subject to two constraints: threshold of motion and wave breaking. For sediment movement *U*_0_^2^ > *U*_t_^2^, where *U*_t_ is the threshold wave-velocity amplitude to be determined below, which when combined with equations (8a, b) gives *x* < tanh*kh*, Eq. (1a), where *x* = *L*/*L*_t∞_ and *L*_t∞_ = πg(*d*_0_/*U*_t_)^2^/2. The wave-breaking criterion^36^ defines the maximum possible wave steepness as, *H*/*L* ≤ 0.142tanh*kh*, which when combined with *d*_0_ and *L*_t∞_ gives *x* ≥ *A*cosh*kh*, Eq. (1b), where *A* = *d*_0_/0.142*L*_t∞_. It will be shown below, that *A* < 1/2, so if *A* = (*U*_t_*/U*_m_)^2^/2, where *U*_m_ = (0.0355π*gd*_0_)^1/2^ is the maximum wave-velocity amplitude (*U*_m_ > *U*_t_), then *U*_t_ < *U*_0_ ≤ *U*_m_ and from Eq. (8b) π*d*_0_/*U*_m_ ≤ *T* <  π*d*_0_/*U*_t_.

The limits of the possible values of *x* can be found by combining Eqs. (1a, b) using the identity 1–tanh^2^*kh* = sech^2^*kh*, such that the maximum and minimum in *x* satisfy the equation *x*^4^–*x*^2^ + *A*^2^ = 0, so that *x*_max, min_ are

9 $${x_{\hbox{max} ,\hbox{min} }}={[1/2 \pm {(1 - 4{A^2})^{1/2}/2}]^{1/2}},$$

where *A* < 1/2, for there to be two distinct values. Here, *x* and *kh* are in the ranges *x*_min_ ≤ *x* < *x*_max_ and arctanh*x* < *kh* ≤ arccosh(*x*/*A*), respectively. In Fig. 1, for the limiting *A* = 1/2 (*U*_m_^2^ = *U*_t_^2^) single-valued case the dot corresponds to *x* = 2^–1/2^, from Eq. (9), and *kh* = arctanh2^–1/2^ ~ 0.88. Likewise, in the *A* = 1/4 (*U*_m_^2^ = 2*U*_t_^2^) case the dots mark *x*_max, min_ = (2 ± 3^1/2^)^1/2^/2~ 0.97, 0.26 and *kh*_max, min_ = arctanh(*x*_max, min_).

### Determination of *U*_t_^2^ based on Soulsby^28^

According to Soulsby^28^, the Shields parameter for the critical threshold of motion of clean sand is

10 $${\uptheta _0}=\frac{{0.3}}{{1+1.2{D_*}}}+0.055(1-{\text{e}^{-0.02D*}}),$$

where D_*_ = [(*s*–1)*g*/ν^2^]^1/3^*D*_50_, *s* = ρ_s_*/*ρ, ρ_s_ and ρ are the sediment and water densities and ν is the kinematic viscosity (~ 1 mm^2^ s^-1^). For waves, θ_0_ = *f*_w_*U*_t_^2^/2(*s*–1)*gD*_50_, where *f*_w_ = 1.39(6*d*_0_/*D*_50_)^–0.52^ is the skin friction factor^37^. Rearranging the θ_0_ wave expression gives *U*_t_^2^ as

11 $$U_{\text{t}}^{{~2}}=~B(gd_{0}^{{~0.52}}D_{{50}}^{{~0.48}}),$$

where *B* = 6^0.52^(*s*–1)θ_0_/0.695 = 3.653(*s*–1)θ_0_. Equation (11) can be compared with Eq. (12).

### Diem^14^ threshold of motion constraint

**Fig. 4:**
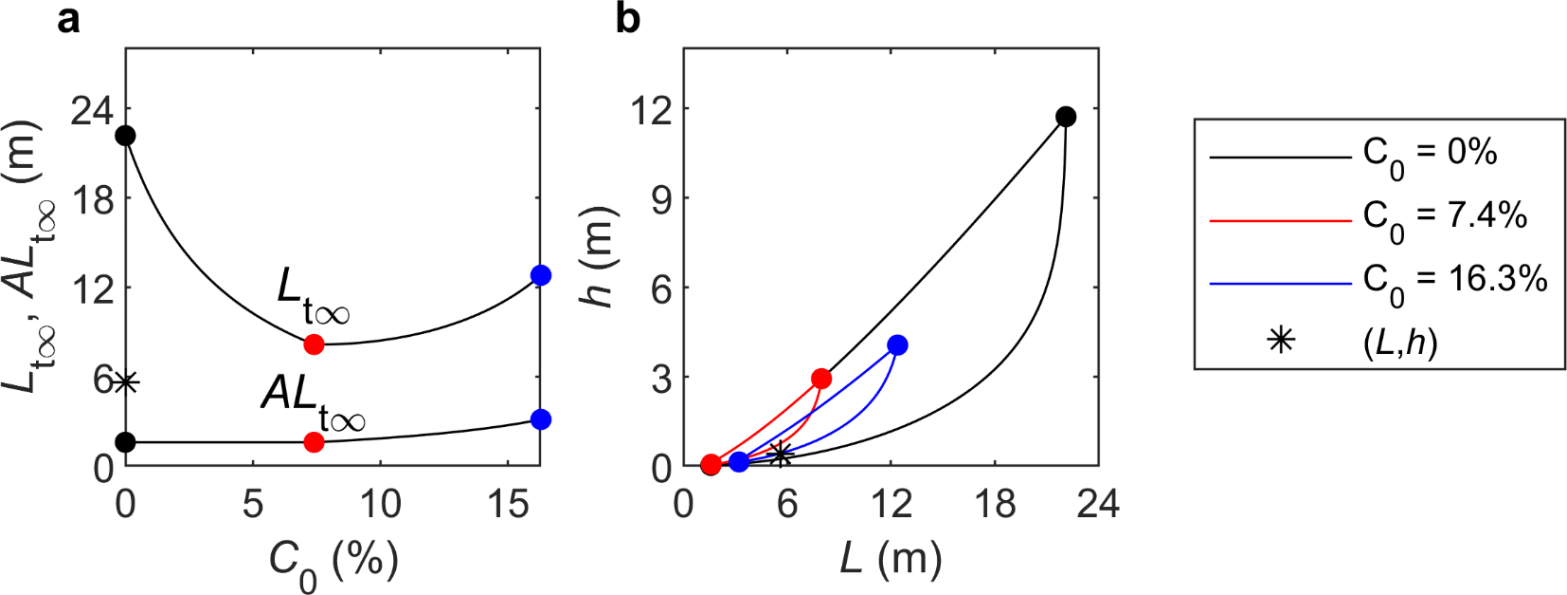
(**a**) Threshold, *L*_t∞_, and wave-breaking scales, *AL*_t∞_, Eqs. (13) and (7c), versus *C*_0_ and (**b**) *L*–*h* phase space from Eq. (2) showing the different ranges for *C*_0_ = 0, 7.4 and 16.3% and the measured *h* and *L* for Wu et al.^11^, λ_e_ = 278*D*_50_, *D*_50_ = 0.496 mm, *h* = 0.6 m and *L* = 5.62 m.

Diem^14^ used the Komar & Miller^29^ expression for *U*_t_^2^, namely

12 $$U_{\text{t}}^{{~2}}=(s - 1)g{d_0}~ \times \left\{ {\begin{array}{*{20}{l}} {0.21{{({d_0}/{D_{50}})}^{ - 0.5}},}&{{D_{50}}<0.5~\;{\text{mm}},} \\ {0.46\uppi {{({d_0}/{D_{50}})}^{ - 0.75}},}&{{D_{50}} \geqslant 0.5~\;{\text{mm}},} \end{array}} \right.$$

such that with the inclusion of clay *L*_t∞_ = π*gd*_0_^2^/2*B*_θ_*U*_t_^2^, *d*_0_ = λ_e_/α and *B*_θ_ and α are given by Eqs. (5) and (6), giving *L*_t∞_ as

13 $${L_{\text{t}\infty }}=\frac{{\uppi {\uplambda _{\text{e}}}}}{{2(s - 1)}} \times \left\{ {\begin{array}{*{20}{l}} {\frac{1}{{0.21[{B_\theta }{\alpha ^{1.5}}]}}{{\left( {\frac{{{\uplambda _{\text{e}}}}}{{{D_{50}}}}} \right)}^{0.5}},}&{{D_{50}}<0.5\;~{\text{mm}},} \\ {\frac{1}{{0.46\uppi [{B_\theta }{\alpha ^{1.75}}]}}{{\left( {\frac{{{\uplambda _{\text{e}}}}}{{{D_{50}}}}} \right)}^{0.75}},}&{{D_{50}} \geqslant 0.5\;~{\text{mm}},} \end{array}} \right.$$

and *AL*_t∞_ as λ_e_/0.142[α] remains the same. Figure 4 shows the effect of this parameterisation of the threshold of motion for the first example case of Wu et al.^11^ depicted in Fig. 2a, d. Unlike Fig. 2d, the measured values of *h* and *L* are outside the range predicted for *C*_0_ = 7.4%.

### Using the Sleath^21^ expression to predict *d*_0_/λ_e_

This section considers the effect of using the Sleath^21^ expression for *d*_0_/λ_e_ when λ_e_ ≥ 200 mm and applies it to the three examples considered in the “Results” section for the clean-sand case. In the clean-sand case, according to Diem^14^, *d*_0_/λ_e_ can be expressed as

14 $$\frac{{{d_0}}}{{{\uplambda _{\text{e}}}}}=\left\{ {\begin{array}{*{20}{l}} {\upalpha _{0}^{{ - 1}},}&{{\uplambda _{\text{e}}}<200\;~{\text{mm}},} \\ {0.778R_{\text{s}}^{{~0.151}},}&{{\uplambda _{\text{e}}} \geqslant 200~\;{\text{mm}},} \end{array}} \right.$$

where α_0_ was taken to be 0.65, but here is 0.61, see Eq. (6), and *R*_s_ = (*U*_0_*d*_0_/2ν)^1/2^. If λ_e_ < 200 mm, then α_0_^–1^λ_e_ can be substituted for *d*_0_, as explained in the main paper. However, for λ_e_ ≥  200 mm, since the *d*_0_/λ_e_ ratio can change, Diem^14^ showed that an additional step in the calculation was required. The wave-velocity amplitude must still be in the range *U*_t_ < *U*_0_ ≤ *U*_m_, so that *R*_s_ is in the range (*U*_t_*d*_0_/2ν)^1/2^ < *R*_s_ ≤ (*U*_m_*d*_0_/2ν)^1/2^, and therefore from Eq. (14), for λ_e_ ≥  200 mm, *d*_0_/λ_e_ must be in the range 0.778(*U*_t_*d*_0_/2ν)^0.0755^ < *d*_0_/λ_e_ ≤  0.778(*U*_m_*d*_0_/2ν)^0.0755^. From Eq. (11), *U*_t_ = (*Bg*)^0.5^*d*_0_^0.26^*D*_50_^0.24^, where *B* = 3.653(*s*–1)θ_0_, and *U*_m_ = (0.0355π*gd*_0_)^0.5^, so that the *d*_0_/λ_e_ range is

15 $${P_1}{\left( {\frac{{{g^{0.5}}d_{0}^{{~1.26}}D_{{50}}^{{~0.24}}}}{\upnu }} \right)^{0.0755}}<~\frac{{{d_0}}}{{{\uplambda _{\text{e}}}}}~ \leqslant ~{P_2}{\left( {\frac{{gd_{0}^{{~~3}}}}{{{\upnu ^2}}}} \right)^{0.03775}},$$

where *P*_1_ = 0.778(*B*/4)^0.03775^, *P*_2_ = 0.778(0.0355π/4)^0.03775^, and the minimum and maximum in the *d*_0_/λ_e_ range correspond to the threshold of motion and wave breaking, respectively. For a given measured λ_e_, the solution to Eq. (15) requires an iteration starting from *d*_0_ = α_0_^–1^λ_e_ = 1.64λ_e_. Substituting λ_e_(*d*_0_/λ_e_)_min_ and λ_e_(*d*_0_/λ_e_)_max_, from Eq. (15), into equations (4a, b,c) allows the threshold of motion and wave breaking scales, *L*_t∞_ and *AL*_t∞_, to be expressed as

16a $${L_{\text{t}\infty }}~=\frac{{\uppi {\uplambda _{\text{e}}}({d_0}/{\uplambda _{\text{e}}})_{{\hbox{min} }}^{{1.48}}}}{{2B}}{\left( {\frac{{{\uplambda _{\text{e}}}}}{{{D_{50}}}}} \right)^{0.48}}$$

16b $$A{L_{\text{t}\infty }}~=\frac{{{\uplambda _{\text{e}}}{{({d_0}/{\uplambda _{\text{e}}})}_{\hbox{max} }}}}{{0.142}}.$$

For each of the three example cases considered in the “Results” section, (*d*_0_/λ_e_)_min_ and (*d*_0_/λ_e_)_max_ are listed in Table 1, even though the λ_e_ ≥ 200 mm condition is only met in the Doucette^32^ case. In all three example cases, *d*_0_/λ_e_ = 1.64 lies between (*d*_0_/λ_e_)_min_ and (*d*_0_/λ_e_)_max_. The observed and predicted *d*_0_/λ_e_ are in close agreement, apart from the Doucette^32^ case, where both the *d*_0_/λ_*e*_ range from Eq. (15) and *d*_0_/λ_e_ = 1.64 underpredict by approximately a factor of two. The values of *L*_t∞R_ and *AL*_t∞R_ from Eqs. (16a, b) and (4b, c) are also given in Table 1. Since these values for *L*_t∞R_ and *AL*_t∞R_ are largely similar in all three cases, this suggests that using the orbital approximation *d*_0_/λ_e_ = 1.64 for the Doucette^32^ case is reasonable, even though λ_e_ ≥  200 mm.

**Table 1:** Measured *D*_50_, λ_e_ and *d*_0_/λ_e_ and predicted θ_0_ from Eq. (10), (*d*_0_/λ_e_)_min_ and (*d*_0_/λ_e_)_max_ from Eq. (15), *L*_t∞R_ = [0.61(*d*_0_/λ_e_)_min_]^1.48^ and *AL*_t∞R_ = 0.61(*d*_0_/λ_e_)_max_, based on Eqs. (16a, b) and (4b, c) for the three example cases.

Data	D_50_ (mm)	λ_e_ (mm)	d_0_/λ_e_ (–)	θ_0_ (–)	(d_0_/λ_e_)_min_ (–)	(d_0_/λ_e_)_max_ (–)	L_t∞*R*_ (–)	AL_t∞*R*_ (–)
Wu et al.^11^	0.496	138	1.64	0.032	1.04	1.79	0.51	1.09
Doucette^32^	0.22	250	3.16	0.045	1.07	1.94	0.53	1.18
Boyd et al.^33^	0.11	180	1.74	0.076	1.07	1.86	0.53	1.13

## Data Availability

All data generated or analysed during this study are included in this published article.
